# A case report on a novel use of intraoperative Intrabeam™ radiation therapy for a recurrent malignant peripheral nerve sheath tumor with sciatic nerve involvement

**DOI:** 10.1186/s13014-022-02135-x

**Published:** 2022-11-18

**Authors:** Edwin Chaharbakhshi, Joshua Hardham, Ramon Alfredo Siochi, Todd C. Tenenholz, Brock A. Lindsey

**Affiliations:** 1 Department of Orthopaedics, West Virginia University, PO Box 9196, 26506-9196 Morgantown, WV USA; 2 WVU School of Medicine, West Virginia University, Morgantown, WV USA; 3 Radiation Oncology, West Virginia University, Morgantown, US

**Keywords:** Malignant peripheral nerve sheath tumor, Intrabeam™ radiation therapy, Local recurrence

## Abstract

**Background:**

Malignant peripheral nerve sheath tumors (MPNST) are sarcomas that arise from peripheral nerves. They generally have a poor prognosis which is exacerbated by high local recurrence rates. This case report discusses the treatment of a patient with a MPNST with local recurrence. This case report is novel due to the use of intraoperative Intrabeam™ (Zeiss, Dublin, CA) radiation therapy use in the protection of neurovascular structures such as the sciatic nerve.

**Case presentation:**

The patient was a 65-year-old male who noticed a right posterior thigh mass slowly increasing in size over two months. A planned positive margin wide-resection excision was performed due to sciatic nerve abutment. The mass was determined to be a MPNST via postoperative pathology with positive margins along the sciatic nerve. The patient began adjuvant radiation therapy to the upper and lower thigh fields over a period of three months. Thirty-two months later, the patient was found to have a hypermetabolic mass with venous congestion and hyperemia at the prior surgical site which was confirmed by core needle biopsy to be local recurrence of the MPNST. Re-excision of the tumor was planned and performed followed by intraoperative Intrabeam™ radiation therapy. At two years of follow-up, the patient was doing well with minimal pain in his right buttock region with no new or recurrent neurological deficits. Radiologic imaging was negative for local recurrence of the MPNST.

**Conclusion:**

We believe this case report demonstrates a novel treatment strategy for sarcoma management. The unique use of intraoperative Intrabeam™ radiation therapy, which had not previously been used for this indication, may be efficacious in cases involving neurovascular structures. In this case, focal radiation from the intraoperative Intrabeam™ radiation device was used in a way to affect the recurrent tumor yet protect the sciatic nerve.

## Background

Malignant peripheral nerve sheath tumors (MPNSTs) are sarcomas that arise from peripheral nerves, accounting for up to 10% of all sarcomas [1]. Up to 60% of cases are associated with neurofibromatosis type 1, with the rest being related to exposure to radiation or sporadic mutation of tumor suppressor genes [1, 2]. Unfortunately, the vast majority of MPNSTs to date do not have a highly specific recognized genetic sequence [3]. Among all sarcoma subtypes, MPNSTs have the highest local recurrence rate. MPNSTs generally have a poor prognosis which is exacerbated by local recurrence or metastasis [4]. This case report summarizes the diagnosis and management of a patient with primary MPNST with local recurrence after planned positive margin wide-resection due to involvement of the sciatic nerve. The local recurrence was managed by re-excision and intraoperative Intrabeam™ (Zeiss, Dublin, CA) radiation therapy, which is traditionally utilized for breast cancer.

## Case presentation

The patient initially presented to clinic as a 65-year-old male who noticed a right posterior thigh mass slowly increasing in size over two months. Although the mass was initially painless, it became painful as it increased in size particularly when sitting or lying down at night. Upon initial presentation, he did not exhibit any numbness or tingling in the right leg. There were no constitutional symptoms present including weight changes, fevers, chills, or night sweats. Four years prior to his presentation, he underwent an uneventful right total knee arthroplasty for osteoarthritis; otherwise, there was no history of prior trauma or pelvic surgeries. The patient’s social history was significant for 50 pack years of smoking. His past medical history was significant for hypertension, benign prostatic hyperplasia, and diabetes complicated by peripheral neuropathy. His family history was significant for pancreatic cancer, lung cancer, and leukemia. There was no personal or family history of neurofibromatosis.

**Fig. 1:**
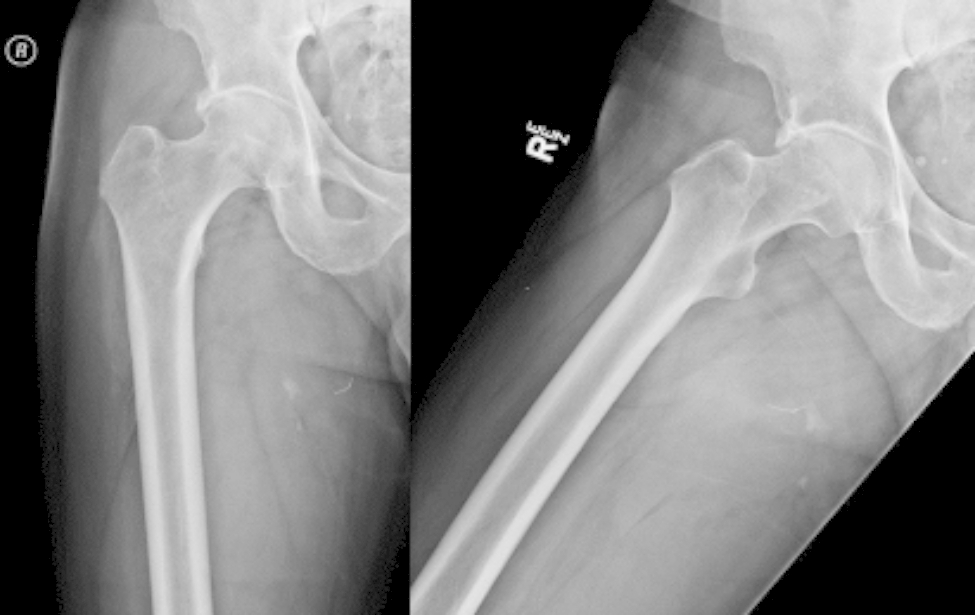
Initial anterior-posterior (left) and lateral (right) radiographs of the right hip

**Fig. 2:**
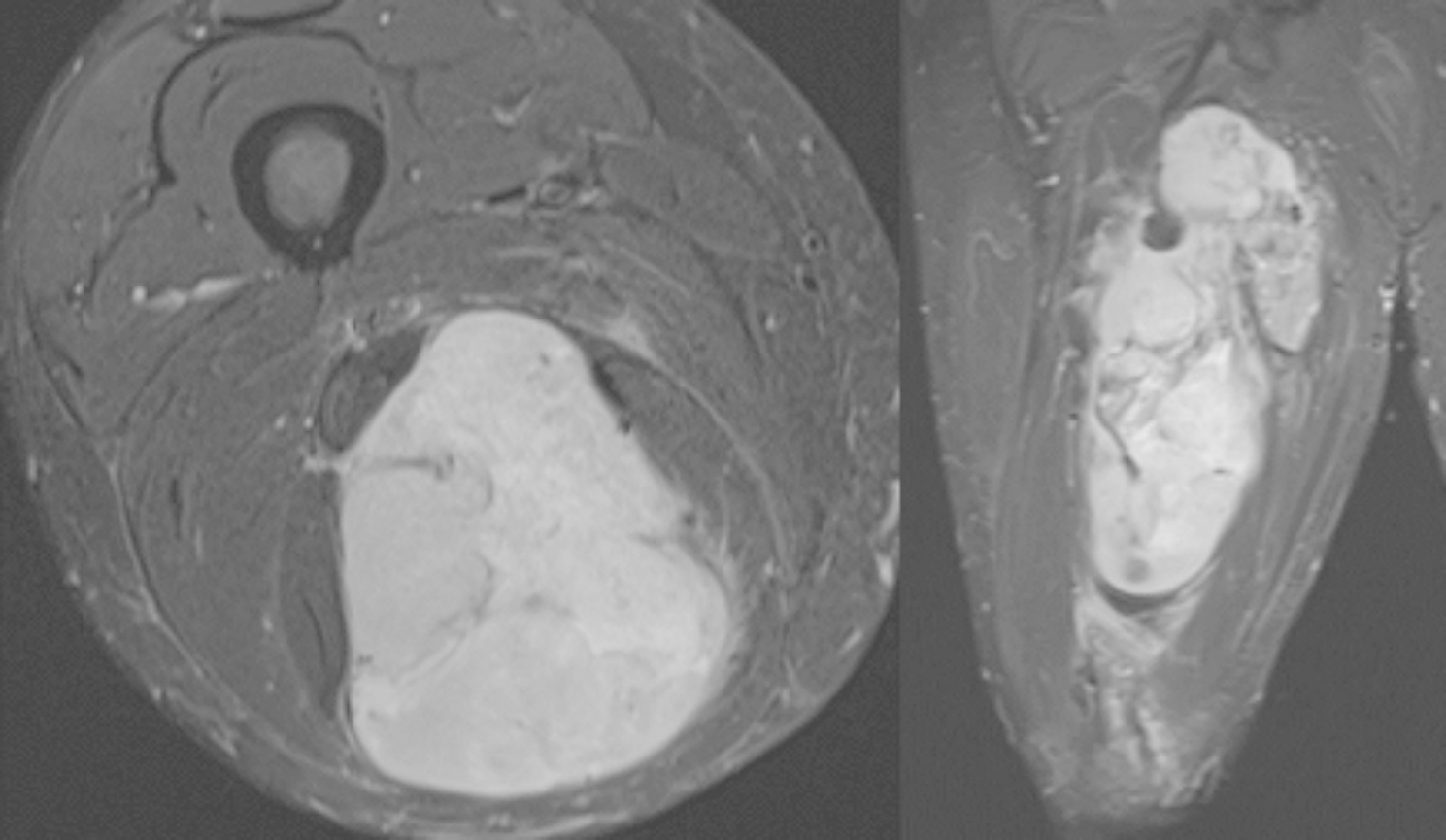
Preoperative T2-weighted MRI (left: axial; right: coronal)

On initial examination, he was found to have a large palpable mass extending from his buttock down to just proximal to his popliteal fossa, which was also visible as a heterogenic soft tissue mass on radiographic imaging of the femur (Fig. 1). The patient had decreased sensation on the plantar surface of his feet from diabetic neuropathy; the examination was otherwise unremarkable. Computed tomography (CT) imaging of the chest was negative for pulmonary lesions. Suboptimal magnetic resonance imaging (MRI) was taken on the day of presentation thus was difficult to interpret, but the initial suspicion was a soft tissue sarcoma. Given his symptoms and initial imaging findings, it was recommended that he undergo a core needle biopsy and an MRI of the femur to better characterize the mass for potential excision. The MRI demonstrated a 24 × 11 × 10 cm heterogeneous mass in the posterior thigh with splaying of the surrounding musculature, initially suggesting a liposarcoma (Fig. 2). Pathology results revealed that it was a high-grade spindle cell sarcoma, with postoperative pathology demonstrating that it was a MPNST with positive margins along the sciatic nerve. The tumor was staged as stage III, PT2b, cN0, cM0.

The tumor was removed via surgical amputation of the semimembranosus, semitendinosus, fascia of the biceps femoris, and abductors. The sciatic nerve and its epineurium were carefully peeled away from the fascia of the biceps femoris, allowing removal of the tumor. Positive margins were planned due to the positioning of the sciatic nerve within the tumor. The patient had mild paresthesias postoperatively in the right foot that resolved spontaneously resulting in a short, uneventful hospital stay. As planned, the superior, anterior, and posterior surgical margins were positive for tumor. The patient returned to clinic two weeks postoperatively, with an appropriately healing wound and no neurovascular deficits. At approximately five weeks postoperatively, the patient began adjuvant radiation therapy in an effort to improve local control and survival. He received 66 grays (Gy) in 33 fractions to the upper and lower thigh fields over a period of three months.

**Fig. 3:**
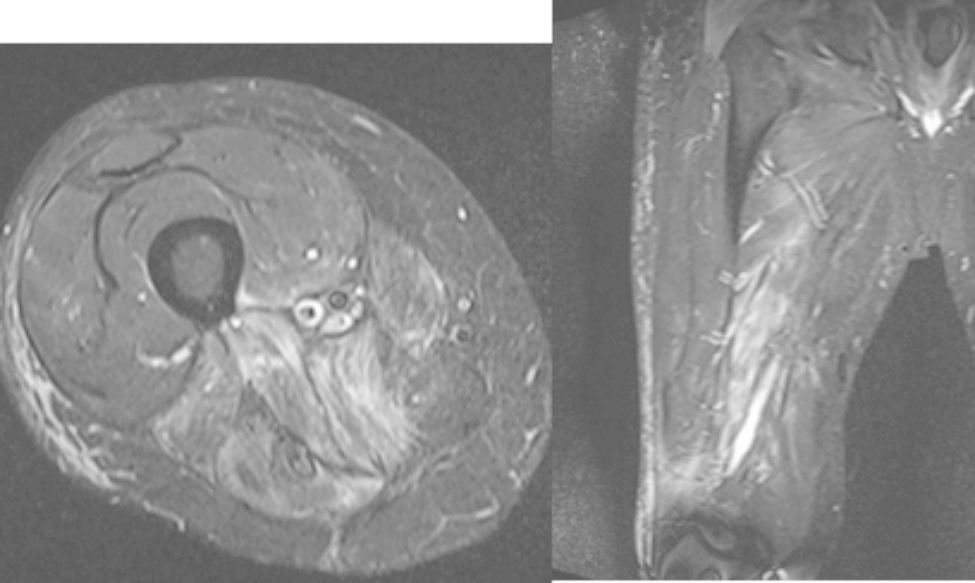
Postoperative T2-weighted MRI (left: axial; right: coronal)

**Fig. 4:**
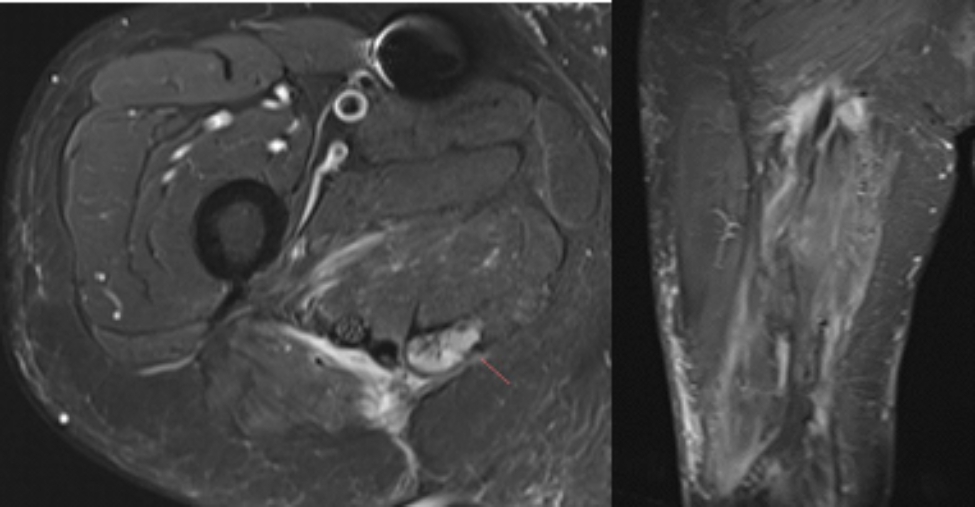
Post-recurrence preoperative T2-weighted MRI (left: axial; right: coronal

The patient was followed with routine positron emission tomography (PET), MRI, and CT scans for surveillance (Fig. 3). Thirty-two months after completion of radiation, the patient was found to have a hypermetabolic mass with venous congestion and hyperemia at the prior surgical site. At this time, the patient had new complaints of worsening pain in his right buttock area and paresthesia down his entire right leg. An MRI demonstrated a 2 cm nodule over the proximal aspect of the right buttock area (Fig. 4). Core needle biopsy confirmed local recurrence of the MPNST.

Surgical excision of the recurrent mass was planned and performed, followed by intraoperative Intrabeam™ radiation therapy. This device is primarily utilized in breast cancer treatment and has a 4.0 cm spherical applicator. The dose using megavoltage energy is 10 Gy for a single fraction. However, at 50 kV (the energy of the Intrabeam™), there is a higher radiobiological effective dose that needs to be considered. The drastically reduced penetration depth compared to 6 mV means that one would be delivering a lower dose at a given distance from the surface applicator. Based on these factors, a clinical decision was made to use 12 Gy. At this dose, the center of the applicator was considered to be 2.5 cm from critical structures which provided a dose of 1 Gy to the tumor. This dose was considered acceptable due to a steep dose fall-off gradient intended to spare the sciatic nerve. The applicator was positioned at least 2 cm from the sciatic nerve and 3 cm from the ischial tuberosity. A dose of 12 Gy (using calibration mode v 4.0) at the surface of the applicator was administered over a period of 13 min. The applicator was then removed, and the surgical wound was closed.

**Fig. 5:**
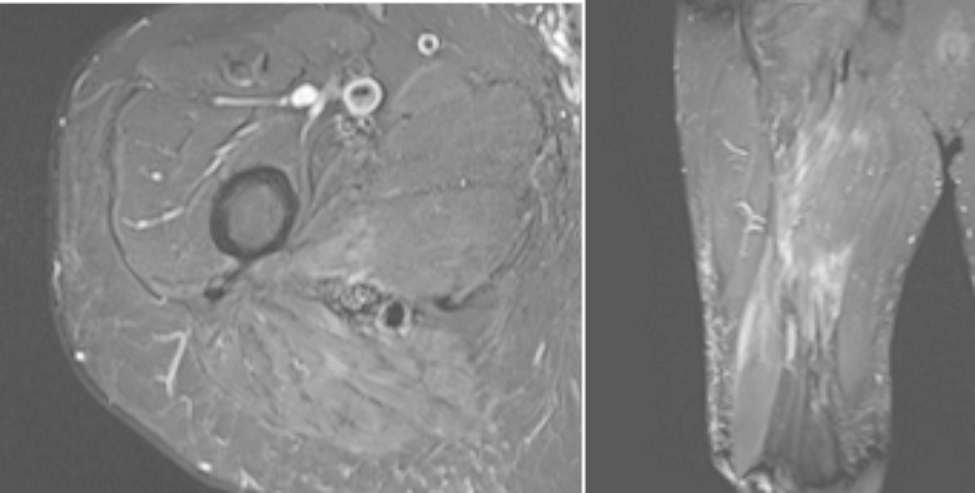
Post-recurrence postoperative T2-weighted MRI (left: axial; right: coronal)

At three weeks postoperatively, the patient had a partially dehisced surgical wound that had small amounts of serous drainage but no clinical signs of infection. The wound was managed with wound vacuum therapy, Dakin’s solution, and a short course of oral antibiotics for prophylaxis. The wound took approximately 15 months to heal using wet to dry dressings. The patient developed cellulitis around the wound which was treated successfully with clindamycin. At two-year follow-up, the patient was doing well with minimal pain in his right buttock region with no new or recurrent neurological deficits. His wound was fully closed with no evidence of infection. Examination and imaging were negative for recurrence at two and a half years postoperatively. (Fig. 5).

## Discussion and conclusions

We believe this case report demonstrates a novel treatment strategy for sarcoma management. Although positive margins predicate local recurrence, a planned positive resection of a MPNST may allow the surgeon to protect neurovascular structures, i.e., the sciatic nerve in this case. Secondly, intraoperative Intrabeam™ radiation therapy, which is not traditionally used for sarcomas, may be efficacious in cases involving neurovascular structures based on previous studies showing a reduction of radiotherapy complications and sparing of critical tissue [5, 6]. Aspects to consider in utilizing this technology are the size and placement of the applicators. In this case, we utilized the 4 cm spherical applicator for treating the MPNST using low-energy x-rays that have a steep dose fall-off gradient, reducing damage to the sciatic nerve. We derived the dosage based on clinical experience with sarcomas treated with external beam radiotherapy. While the intraoperative Intrabeam™ radiation device has been used for indications outside breast cancer, the dosage was derived from previous experience with sarcomas with the intent of protecting the sciatic nerve [7, 8]. Given MPNSTs are typically associated with poor prognosis, especially in recurrent cases, novel treatment strategies are desired by oncologists. This case report demonstrates a novel use of focal radiation therapy using intraoperative Intrabeam™ radiation therapy in a way that affected the recurrent tumor yet protected the sciatic nerve. However, further research into protecting neurovascular structures while using intraoperative Intrabeam™ radiation therapy should be performed as this case report is limited to a single patient.

## Data Availability

Data sharing is not applicable to this article as no datasets were generated or analyzed during the current study.

## List of abbreviations

MPNST: Malignant peripheral nerve sheath tumors
Gy: Grays

## References

[1] Mohamad T, Plante C, Brosseau J-P. Toward Understanding the Mechanisms of Malignant Peripheral Nerve Sheath Tumor Development. Int J Mol Sci. 2021 Aug 10;22(16):8620. 10.3390/ijms22168620 PMC8395254 34445326

[2] James AW, Shurell E, Singh A, Dry SM, Eilber FC (2016). Malignant Peripheral Nerve Sheath Tumor. Surg Oncol Clin N Am.

[3] Thway K, Fisher C (2014). Malignant peripheral nerve sheath tumor: pathology and genetics. Ann Diagn Pathol.

[4] Cai Z, Tang X, Liang H, Yang R, Yan T, Guo W (2020). Prognosis and risk factors for malignant peripheral nerve sheath tumor: a systematic review and meta-analysis. World J Surg Oncol.

[5] Song X, Shao Z, Liang H. Using the new INTRABEAM mobile intraoperative radiotherapy system during surgery for pancreatic cancer: a case report. J Med Case Rep. 2019 Jan 26;13(1):23. doi: 10.1186/s13256-018-1906-6. PMID: 30683151; PMCID: PMC6347751. 10.1186/s13256-018-1906-6 PMC6347751 30683151

[6] Pan L, Ye C, Chen L, Tang W, Zhang X, Gao J, Wu R, Ye X, Tan W, Wan M, Zheng W (2019). Oncologic outcomes and radiation safety of nipple-sparing mastectomy with intraoperative radiotherapy for breast cancer. Breast Cancer.

[7] Cui TX, Dai JG, Li JM, Qian JD, Li GH, Sun JG (2020). Safety and efficacy of INTRABEAM intraoperative radiotherapy for invasive thymoma. Med (Baltim).

[8] Rahy-Martín AC, Cruz-Benavides F, Sánchez-Lauro M (2018). Intraoperative radiotherapy with the Intrabeam^®^ device for the treatment of resectable pancreatic adenocarcinoma. Radioterapia intraoperatoria con Intrabeam^®^ para el tratamiento del adenocarcinoma de páncreas resecable. Cir Esp (Engl Ed).

